# Impact of Clinical Pharmacist Interventions on Medication Safety and Therapy Optimization in a Tertiary Care Hospital

**DOI:** 10.7759/cureus.104661

**Published:** 2026-03-04

**Authors:** Sana Rahim

**Affiliations:** 1 Clinical Pharmacy, Darul Sehat Hospital, Karachi, PAK

**Keywords:** clinical pharmacist interventions, critical care pharmacy, dose optimization, drug-drug interactions, drug-related problems, inpatient medication review, medication safety, medication therapy optimization, prescriber acceptance, quality improvement

## Abstract

Background

Medication-related problems remain a significant concern in hospital-based care, particularly in critically ill and pediatric populations where pharmacotherapy is complex and frequently modified. Clinical pharmacist-led medication review may improve medication safety through structured identification of therapy-related issues.

Methodology

This retrospective quality improvement evaluation analyzed structured inpatient medication review encounters conducted over a 14-month period at a tertiary care hospital. Intervention frequency, category distribution, and prescriber acceptance were assessed. Descriptive statistics were calculated, and chi-square testing was performed to evaluate associations between intervention category and acceptance status.

Results

Over 5,800 medication review encounters resulted in 2,441 clinical interventions. Dose optimization constituted the largest intervention category. The overall acceptance rate was 2,212 (90.6%). A statistically significant association was observed between intervention category and acceptance status (χ² = 46.46, df = 5, p < 0.001).

Conclusions

Structured clinical pharmacist involvement resulted in frequent and widely accepted interventions during inpatient medication review. Significant variability in acceptance patterns across intervention categories supports the clinical integration of pharmacist services within tertiary care hospital settings.

## Introduction

Medication-related problems remain a significant concern in hospital-based care and are associated with increased morbidity, prolonged hospitalization, and higher healthcare costs [1-4]. Hospitalized patients, particularly those in intensive care and pediatric settings, are exposed to complex pharmacotherapy regimens requiring frequent dose adjustments, monitoring of laboratory parameters, and evaluation for potential drug-drug interactions [1,5,6]. Studies across multiple healthcare systems have demonstrated that drug-related problems occur in a substantial proportion of inpatients and frequently require active intervention to prevent adverse outcomes [2,3,7].

Clinical pharmacists play a critical role in identifying and resolving medication-related problems through structured medication review and interdisciplinary collaboration [5-8]. Participation of pharmacists in multidisciplinary care teams, particularly in critical care settings, has been associated with improved prescribing practices, reduction in adverse drug events, and high rates of prescriber acceptance of pharmacist recommendations [5,8,9]. Intervention-based studies have shown that pharmacist-led medication review consistently identifies opportunities for dose optimization, therapy modification, and interaction management [7,10,11].

Despite growing international evidence supporting clinical pharmacy services, published data from tertiary care hospitals in Pakistan remain limited. Descriptive evaluations of routine pharmacist activity in local settings are necessary to characterize intervention patterns and demonstrate integration within inpatient workflows. This study, therefore, evaluates structured clinical pharmacist activity over a 14-month period in a tertiary care hospital, with the objective of quantifying medication-related problems identified during inpatient medication review and assessing the scope and acceptance of pharmacist-led safety interventions aimed at optimizing medication therapy.

## Materials and methods

Study design

This study was conducted as a retrospective quality improvement evaluation of routine clinical pharmacist activity.

Study setting

The evaluation was conducted at Darul Sehat Hospital, Karachi, Pakistan, a tertiary care facility providing medical, pediatric, surgical, and critical care services. During the study period, the clinical pharmacist was primarily assigned to intensive care units, with additional coverage provided to general medical, pediatric, surgical, and private or semi-private wards [5,6].

Study period

The study was conducted from February 2022 through March 2023 (14 months).

Definition of medication review encounter

A medication review encounter was defined as a documented daily review of an inpatient’s medication regimen performed by the clinical pharmacist [9]. Patients admitted for multiple days contributed multiple review encounters.

Data collection

All medication review encounters and associated interventions were extracted from prospectively maintained documentation logs. Data were de-identified before analysis.

Intervention classification

Each intervention was categorized based on its primary clinical intent into one mutually exclusive category. When an intervention could reasonably fall into more than one category, it was classified according to the principal clinical problem it addressed.

Medication Selection Optimization

Inappropriate, contraindicated, or non-guideline-concordant drug choice; restricted or banned medications; drug-disease interactions; wrong vaccine selection; or incomplete prescribing requiring selection of a more appropriate pharmacologic agent.

Dose Optimization

Underdosing, overdosing, renal or hepatic dose adjustments, weight-based corrections, and other patient-specific dose modifications.

Administration Optimization

Errors related to route, frequency, infusion rate, dilution, preparation, timing, or technical execution of medication orders.

Therapy Optimization

Recommendations aimed at improving the overall treatment plan rather than substituting a specific agent, including duration correction, duplicate therapy resolution, delayed discontinuation, IV-to-oral conversion when clinically appropriate, escalation or de-escalation of therapy based on clinical status, and modification of therapy intensity.

Drug-Drug Interaction Management

Identification and management of clinically significant pharmacokinetic or pharmacodynamic drug interactions.

Electrolyte and Laboratory-Driven Interventions

Recommendations prompted by abnormal laboratory values, electrolyte disturbances, or therapeutic drug monitoring parameters.

Intervention outcomes were recorded as accepted or not accepted based on documented prescriber action [11-13]. An intervention was classified as “accepted” if the prescribing physician modified the relevant medication order following the pharmacist’s recommendation, as reflected in the patient’s documented treatment plan. Recommendations were communicated directly to the prescriber during clinical workflow and recorded in the patient file. If no corresponding change in medication order occurred, the intervention was classified as “not accepted.”

Statistical analysis

Descriptive statistics were used to summarize medication review encounters, intervention frequency, distribution by care setting, intervention category, and acceptance status. A chi-square test of independence was performed to evaluate the association between intervention category and acceptance status. A p-value <0.05 was considered statistically significant.

## Results

Overall review activity and intervention density

During the 14-month study period, a total of 5,815 medication review encounters were documented, generating 2,441 interventions (42.0 per 100 encounters).

Distribution by inpatient care setting

The distribution of interventions across inpatient care settings is summarized in Table 1. Interventions were most frequently documented in critical care units, reflecting the primary clinical deployment of pharmacist services in intensive care settings.

**Table 1:** Distribution of clinical pharmacist interventions by inpatient care setting (n = 2,441).

Inpatient care setting	Interventions, n (%)
Critical care	1,838 (75.30%)
General medical	314 (12.86%)
Pediatrics	156 (6.39%)
Private/Semi-private	110 (4.51%)
Surgical	23 (0.94%)
Grand total	2,441 (100.00%)

Distribution by intervention category

The distribution of interventions according to intervention category is presented in Table 2. Dose optimization constituted the largest category of interventions, followed by drug-drug interaction management and medication selection optimization.

**Table 2:** Distribution of clinical pharmacist interventions by intervention category.

Intervention category	Interventions, n (%)
Administration optimization	176 (7.21%)
Dose optimization	1,273 (52.15%)
Drug-drug interactions management	454 (18.60%)
Electrolyte and laboratory-driven interventions	131 (5.37%)
Medication selection optimization	378 (15.49%)
Therapy optimization	29 (1.19%)
Grand total	2,441 (100.00%)

Acceptance of interventions

Of the 2,441 documented interventions, 2,212 (90.6%) were accepted, and 229 (9.4%) were not accepted. High acceptance rates were observed across all intervention categories. Acceptance status stratified by intervention category is presented in Table 3.

**Table 3:** Acceptance status of clinical pharmacist interventions by category. Chi-square test of independence: χ² = 46.46, df = 5, p < 0.001.

Intervention category	Accepted, n (%)	Not accepted, n (%)	Total, n (%)
Administration optimization	135 (5.53%)	41 (1.68%)	176 (7.21%)
Dose optimization	1,159 (47.48%)	114 (4.67%)	1,273 (52.15%)
Drug-drug interactions management	417 (17.08%)	37 (1.52%)	454 (18.60%)
Electrolyte and laboratory-driven interventions	119 (4.88%)	12 (0.49%)	131 (5.37%)
Medication selection optimization	354 (14.50%)	24 (0.98%)	378 (15.49%)
Therapy optimization	28 (1.15%)	1 (0.04%)	29 (1.19%)
Grand total	2,212 (90.62%)	229 (9.38%)	2,441 (100.00%)

A chi-square test of independence demonstrated a statistically significant association between intervention category and acceptance status (χ² = 46.46, df = 5, p < 0.001), indicating that acceptance proportions varied across intervention categories. Acceptance ranged from 76.7% for administration optimization to 96.5% for therapy optimization, with the remaining categories demonstrating acceptance proportions between 90% and 94%. Administration optimization contributed the largest component to the overall chi-square statistic.

## Discussion

This quality improvement evaluation demonstrates structured and sustained pharmacist-led medication review activity in a tertiary care hospital setting. Over 5,800 documented medication review encounters resulted in 2,441 clinical interventions, reflecting consistent identification of medication-related problems during routine inpatient care [1,7].

Dose optimization constituted the largest proportion of interventions, which is consistent with the complexity of pharmacotherapy in critically ill and pediatric populations, where frequent dose adjustments are required [1]. In the present study, dose modifications were prompted by a combination of renal impairment, hepatic dysfunction, acute organ failure, therapeutic drug monitoring requirements, geriatric pharmacokinetic considerations, and pediatric weight-based dosing. The concentration of interventions within intensive care units reflects both the higher acuity of patients in these settings and the primary clinical deployment of pharmacist services in critical care environments [5,6,14,15].

An intervention density of 42 interventions per 100 medication review encounters provides a measurable indicator of pharmacist engagement within daily inpatient workflows. This process-based metric highlights the frequency with which structured medication review identifies opportunities for therapy modification and safety optimization [10,16].

The overall acceptance rate of 2,212 (90.6%) suggests strong integration of the clinical pharmacist within the multidisciplinary care team and supports the clinical relevance of pharmacist-generated recommendations [12,13,17,18]. A statistically significant association between intervention category and acceptance status indicates that prescriber acceptance varied across different types of interventions. Although acceptance was high across all categories, the observed variability suggests that certain intervention types were more readily incorporated into clinical decision-making than others, reinforcing the collaborative nature of inpatient medication management.

The statistically significant association between intervention category and acceptance status suggests that prescriber responsiveness varies depending on the clinical nature of the recommendation. Interventions involving dose optimization and therapy modification demonstrated higher acceptance proportions, potentially reflecting clearer guideline-driven decision frameworks in these domains, whereas administration-related interventions demonstrated comparatively lower acceptance, possibly due to workflow or practice preference considerations.

Although overall acceptance was high, 9.4% of interventions were not implemented. Potential contributors to non-acceptance may include differences in clinical judgment, evolving patient-specific considerations, and workflow constraints in high-acuity settings. Future efforts to enhance implementation may include structured documentation systems, interdisciplinary case review mechanisms, and prospective evaluation of factors influencing implementation of pharmacist recommendations.

It is important to acknowledge several limitations when interpreting these findings. Individual patients could contribute multiple medication review encounters due to routine daily review practices. Pharmacist deployment was primarily concentrated in intensive care units, which may influence the distribution of interventions across hospital settings. The study was conducted at a single center with one clinical pharmacist, which may limit generalizability. Additionally, findings reflect documented interventions and may not capture informal or undocumented clinical discussions.

Because interventions were identified, categorized, and logged by a single clinical pharmacist without independent adjudication, the study is subject to potential self-reporting bias. Although acceptance required documented prescriber modification of medication orders, the clinical appropriateness of interventions was not independently reviewed. Furthermore, the study evaluated process-based metrics rather than downstream patient-centered clinical outcomes such as adverse drug events, length of stay, mortality, or cost-effectiveness.

## Conclusions

This quality improvement evaluation demonstrates that structured clinical pharmacist-led medication review is associated with consistent identification of medication-related problems and high levels of prescriber acceptance in a tertiary care hospital setting. The results highlight the clinical value of integrating pharmacists into routine inpatient workflows, particularly in complex care environments where pharmacotherapy frequently requires optimization. Although statistically significant variability in acceptance was observed across intervention categories, overall prescriber acceptance remained high. This variability suggests that the nature of clinical recommendations may influence implementation within multidisciplinary teams. Continued incorporation and refinement of structured clinical pharmacy services may contribute meaningfully to medication safety efforts and therapy optimization in hospital-based care.
